# Post-Quantum Secure Identity-Based Proxy Blind Signature Scheme on a Lattice

**DOI:** 10.3390/e25081157

**Published:** 2023-08-02

**Authors:** Fengyin Li, Mengjiao Yang, Zhihao Song, Ping Wang, Guoping Li

**Affiliations:** School of Computer Science, Qufu Normal University, Rizhao 276800, China; ymj5859@163.com (M.Y.); szh00003499@163.com (Z.S.); wang_00_ping@163.com (P.W.); 19819513250@163.com (G.L.)

**Keywords:** blind signature, quantum attack, identity, proxy blind signature, e-voting

## Abstract

Blind signatures have been widely applied when privacy preserving is required, and the delegation of blind signature rights and a proxy blind signature (Proxy-BS) become necessary when the signer cannot sign. Existing Proxy-BS schemes are based on traditional cryptographically hard problems, and they cannot resist quantum attacks. Moreover, most current Proxy-BS schemes depend on public key infrastructure (PKI), which leads to high certificate storage and management overhead. To simplify key management and resist quantum attacks, we propose a post-quantum secure identity-based proxy blind signature (ID-Proxy-BS) scheme on a lattice using a matrix cascade technique and lattice cryptosystem. Under the random oracle model (ROM), the security of the proposed scheme is proved. Security shows that the proposed scheme assures security against quantum attacks and satisfies the correctness, blindness, and unforgeability. In addition, we apply the ID-Proxy-BS scheme on a lattice to e-voting and propose a quantum-resistant proxy e-voting system, which is resistant to quantum attacks and achieves the efficiency of e-voting.

## 1. Introduction

A proxy blind signature (Proxy-BS) is a peculiar type of digital signature and is widely applied in e-government systems [1]. Proxy-BS was first proposed by Lin et al. [2]. It allows the original signer to grant their binding signing rights to the proxy signer (P-signer), after which the P-signer signs without revealing the context of the signed message. Therefore, the two properties of Proxy-BS, namely blindness and unforgeability [3,4], guarantee the privacy of the message and security of the signature. Subsequently, a large number of Proxy-BS schemes based on public key cryptography have been proposed. The RSA-based Proxy-BS scheme [5], Proxy-BS scheme based on DLP and ECDLP [6], and Schnorr-based Proxy-BS scheme [7] have been proposed.

However, with the advent of quantum computers, traditional signature schemes such as RSA and DSA have become insecure since the probabilistic polynomial time algorithm was proposed by Shor [8]. Therefore, the lattice-based signature algorithm is one of the most promising candidate technologies. In 1996, AJTAI proposed a lattice-based cryptographic scheme and proved that it is resistant to quantum attacks [9]. Subsequently, a signature scheme based on NTRU was proposed, but it was soon broken by Regev et al. [10,11]. In 2008, Gentry et al. constructed a GPV signature scheme and proved that it satisfies security under the ROM [12]. In 2013, Ducas et al. proposed a new no-sampling algorithm that samples from a bimodal Gaussian distribution and proposed a lattice signing scheme based on this new no-sampling algorithm [13]. In 2014, Zhang et al. proposed a lattice-based Proxy-BS scheme under the standard model and proved its security based on the small integer solution (SIS) [14]. In 2022, Gu et al. proposed device-independent quantum key distribution, which can provide unconditional security for communication between users [15]. In 2023, Yin et al. proposed an experimental secure network, which enables unconditionally secure quantum digital signatures and encryption [16].

The above Proxy-BS schemes are based on the PKI [17]. In the public key cryptosystem based on the PKI, the user’s identity (ID) and public key (pk) are bound through the certificate, which involves cumbersome storage and legality verification of the certificate. As an alternative to the PKI-based public key cryptosystem, in 1984, Shamir took the user’s ID as the user’s pk and proposed the notion of identity encryption. Identity-based cryptography (IBC) also comes from this [18].

In 2017, Gao et al. improved Rückert’s scheme and proposed an identity-based blind signature scheme [19]. In 2018, Ye et al. proposed a partial Proxy-BS scheme, which was constructed based on identity and lattice [20]. Although these blind signature schemes are resistant to quantum attacks, they ignore the problem of master key leakage. In 2021, Zhou et al. proposed a lattice-based partial Proxy-BS scheme, which satisfies security such as resistance to master key disclosure attacks and unforgeability [21]. Proxy-BS can provide proxy delegation and anonymous authentication, preserve the privacy of the user, and is widely applied in e-government and blockchain systems. Therefore, we combined an identity-based cryptosystem with proxy technology on a lattice to design an efficient and quantum-resistant Proxy-BS scheme.

In this paper, we propose a post-quantum secure identity-based proxy blind signature (ID-Proxy-BS) scheme on a lattice. We apply the ID-Proxy-BS scheme to e-voting and design a quantum-resistant proxy e-voting system, which achieves multi-regional e-voting and ensures the anonymity of ballot content in e-voting. The contributions of this study are given below:

- To simplify the key management and resistance to quantum attacks, we propose a post-quantum secure identity-based proxy blind signature (ID-Proxy-BS) scheme on a lattice using a matrix cascade technique and lattice cryptosystem. In the proposed ID-Proxy-BS scheme on a lattice, we cascade user identity and the master public key to construct the public key of the lattice signature and generate random parameters through a bimodal Gaussian distribution and rejection sampling algorithm. The ID-Proxy-BS scheme has better security.
- Under the ROM, the security of the ID-Proxy-BS scheme on a lattice is proved under the assumption of the small integer solution (SIS) problem.
- To achieve efficient e-voting, we apply the ID-Proxy-BS scheme on a lattice to e-voting and design a quantum-resistant proxy e-voting system. The system achieves multi-regional e-voting and ensures the anonymity of ballot content in e-voting.

## 2. Preliminaries

### 2.1. Lattice Theory

In this section, we define the lattice and a hard problem on the lattice. The specific definitions are below:

**Definition** **1** (Lattice)**.** *Let $B=\{b_{1},b_{2},\ldots b_{k}\}$, in which $b_{1},b_{2},\ldots b_{k}\in R^{m}$ are not correlated with each other. Then, the set of linear combinations of $b_{1},b_{2},\ldots b_{k}$ is called lattice *Λ*; that is,*

(1) $$\Lambda =L\left(B\right)=\left\{c_{1}b_{1}+c_{2}b_{2}+\ldots c_{k}b_{k}\left|c_{i}\right.\in Z\right\}$$

*where B is a basis of ∧ [22].*

*Let q be a prime number, matrix $B\in Z_{q}^{n\times m}$ and vector $u\in Z_{q}^{n}$. The q-ary lattice of the matrix B and the coset of the lattice $\Lambda_{q}^{⊥}\left(B\right)$ are defined as follows:*

(2) $$\Lambda_{q}^{⊥}\left(B\right)=\left\{Bx=0\;\mathrm{mod}\;q|x\in Z^{m}\right\}$$

(3) $$\Lambda_{q}^{u}\left(B\right)=\left\{Bx=u\;\mathrm{mod}\;q|x\in Z^{m}\right\}$$

**Definition** **2** (SIS problem)**.** *Given a real number ω, a prime q, and a matrix $A\in Z_{q}^{n\times m}$, we solve a vector $y\in z^{m}$ such that Ay=0modq and ‖y‖≤ω [23].*

**Lemma** **1.** *For arbitrary $A\in Z_{q}^{n\times m}$, m>64+nlogq/log(2d+1), we randomly choose a vector $x\in {\left\{-d,\ldots ,d\right\}}^{m}$, and with probability $1-2^{100}$, we can find another $x^{\prime }\in \{-d,\ldots ,0,\ldots {d\}}^{m}$ that satisfies $Ax=Ax^{\prime }$ [24].*

### 2.2. Statistical Distance

**Definition** **3** (Statistical distance)**.** *Given two random variables U, V∈S, the statistical distance between U and V is given by*

(4) $$\Delta \left(U,V\right)=\frac{1}{2}\sum_{n\in S}\left|\mathrm{Pr}\left[U=u\right]-\mathrm{Pr}\left[V=u\right]\right|$$

*where S is a finite set [13].*

### 2.3. Gaussian Distribution

**Definition** **4.** *Gaussian distribution: For c>0 and σ>0, we have a Gaussian function $\rho_{c,\sigma }\left(x\right)=e^{-\frac{\pi ||x-c|{|}^{2}}{\sigma^{2}}}$ centered on c and parameter σ. Then, for any x∈∧, the Gaussian distribution is $D_{\wedge ,\sigma ,c}\left(x\right)=\frac{\rho_{\sigma ,c}\left(x\right)}{\rho_{\sigma ,c}\left(\wedge \right)}$ [25].*

### 2.4. Trapdoor Generation and Preimage Sampling Algorithm

In this section, two algorithms are mainly introduced, which are the trapdoor generation algorithm and the preimage sampling algorithm [26]. The trapdoor generation algorithm generates a trapdoor of the lattice (i.e., a short base of the lattice), which is usually used as the master private key. The preimage sampling algorithm uses a trapdoor to generate private keys.

**Definition** **5** (Trapdoor Generation Algorithm)**.** *Let q, m, n be positive integers, where q≥2 and m≥nlogq. There exists an algorithm TrapGen(q,m,n) that outputs B and a basis $T\in Z^{m\times m}$ of lattice $\Lambda^{⊥}\left(B\right)$ such that the distribution of $B\in Z_{q}^{n\times m}$ is statistically indistinguishable from the distribution of $Z_{q}^{n\times m}$, and $\left|\right|\tilde{T}\left|\right|\leq O(\sqrt{n\log q})$.*

**Definition** **6** (Preimage Sampling Algorithm)**.** *Given a matrix B, a trapdoor basis T of lattice $\Lambda^{⊥}\left(B\right)$, a target term $u\in Z_{q}^{n}$, and $x\geq \left|\right|\tilde{T}\left|\right|\cdot \omega \left(\sqrt{\log q}\right)$, there exists a polynomial algorithm SamplePre(B,T,x,u) that outputs a vector $y\in \Lambda^{u}\left(B\right)$, and the distribution of y is statistically close to $G_{\Lambda^{u}\left(B\right),x}$.*

## 3. Security Model

The proxy blind signature (Proxy-BS) scheme satisfies the blindness and unforgeability of the signature scheme. Blindness primarily considers adversary signers. An adversary signer cannot find an arbitrary message–signature pair by implementing a specific signature algorithm. Unforgeability considers malicious original signers $F_{1}$. Next, we prove the security of the scheme through games between an adversary signer and a user, adversary $F_{1}$ and the challenger.

### 3.1. Blindness

The blindness is proved through a game ${\mathrm{Game}}_{S}^{blind}$ between an adversary signer and two users.

**Definition** **7** (Blindness)**.** *The scheme satisfies blindness if no adversary S wins the game with non-negligible probability δ. This game ${\mathrm{Game}}_{S}^{blind}$ is below.*

*${\mathrm{Game}}_{S}^{blind}$: $U_{1}$ and $U_{2}$ are two users, S is an adversary. The specific process of this game is as follows:*

*Setup: We have a random coin b∈{0,1}, which cannot be known by S. $U_{1}$ and $U_{2}$ randomly select two messages $m_{b}$ and $m_{1-b}$, respectively, and send them to S.*

*Signature: After S has received the message from $U_{1}$ and $U_{2}$, S executes the blind signature algorithm with two users $U_{1}\left(m_{b}\right)$ and $U_{2}\left(m_{1-b}\right)$ simultaneously, and finally $U_{1}$ and $U_{2}$ generate signatures $\sigma (m_{b})$ and $\sigma (m_{1-b})$, respectively, and send them to S.*

*Guess: After S has received the signature from $U_{1}$ and $U_{2}$, S guesses b.*

*The adversary S’s advantage in winning the above game is $|\mathrm{Pr}\left[Game_{S}^{Bl\mathrm{in}d}=1\right]-\frac{1}{2}|$, where $\mathrm{Pr}[{\mathrm{Game}}_{S}^{Bl\mathrm{in}d}=1]$ is the probability that ${\mathrm{Game}}_{S}^{Bl\mathrm{in}d}=1$.*

### 3.2. Unforgeability

The Proxy-BS scheme satisfies existential unforgeability under adaptive chosen message attack (EUF-CMA). The EUF-CMA security model has a malicious original signer $F_{1}$. $F_{1}$ knows the proxy key, but not the proxy signer’s private key. We demonstrate the security of the Proxy-BS scheme through a game between the adversary and the challenger.

**Definition** **8** (EUF-CMA)**.** *The scheme satisfies EUF-CMA security if no adversary $F_{1}$ wins the game with non-negligible probability δ. This game $\mathrm{Gam}e_{F_{1}}$ is given below.*

*$\mathrm{Gam}e_{F_{1}}$: T is a challenger, $F_{1}$ is an adversary. $F_{1}$ knows the proxy key. The specific process of this game is as follows:*

*Random oracle queries: $F_{1}$ queries the hash value of the message $m_{i}$, and T returns the hash result of $m_{i}$ to $F_{1}$.*

*Signature queries: $F_{1}$ queries the signature of the message $m_{i}$, T returns signature to $F_{1}$.*

*Forge: $F_{1}$ returns a forged signature of a message. If the signature is valid, $F_{1}$ wins the game. The advantage of $F_{1}$ in winning the game is the probability of returning a valid signature.*

## 4. Identity-Based Proxy Blind Signature Model

This section introduces an identity-based proxy blind signature scheme model, which consists of five algorithms (Setup, KeyGen, ProxyKeyGen, Proxy-BS, Verify) [27]. This algorithm is completed by the interaction between the original signer O-signer, the proxy signer P-signer, and the user User. The specific steps are as follows.

1. $\mathrm{Setup}(1^{\lambda })\to pp$: It inputs security parameters and generates system parameters;
2. $\mathrm{KeyGen}\left(pp,ID_{o},ID_{p},\sigma \right)\to S_{o},S_{p}$: It inputs system parameters, public keys of O-signer and P-signer, and generates private keys of O-signer and P-signer;
3. $\mathrm{ProxyKeyGen}(pp,ID_{o},ID_{p},S_{o})\to S$: It inputs system parameters, O-signer’s key pair, and P-signer’s public key, and generates a proxy key;
4. $\mathrm{Proxy}-\mathrm{BS}(pp,S_{p},S,M)\to c$: It inputs system parameters, message, and P-signer’s private key and proxy key, and the algorithm generates a blind signature of the message;
5. $\mathrm{Verify}(pp,ID_{o},ID_{p}M,c)\to 1\;\mathrm{or}\;0$: It inputs a message and its corresponding blind signature; the algorithm verifies that the signature is valid. If it is, the signature is accepted; otherwise, the signature is rejected.

## 5. Identity-Based Proxy Blind Signature (ID-Proxy-BS) Scheme on a Lattice

To achieve the anti-quantum attack performance of the proxy blind signature (Proxy-BS) scheme and solve the certificate management problem of the Proxy-BS scheme, this section proposes an identity-based proxy blind signature (ID-Proxy-BS) scheme on a lattice using a matrix cascade technique and lattice cryptosystem. This scheme cascades user identity and the master public key to construct the public key of the lattice signature, and generates random parameters through a bimodal Gaussian distribution and rejection sampling algorithm.

**Figure 1:**
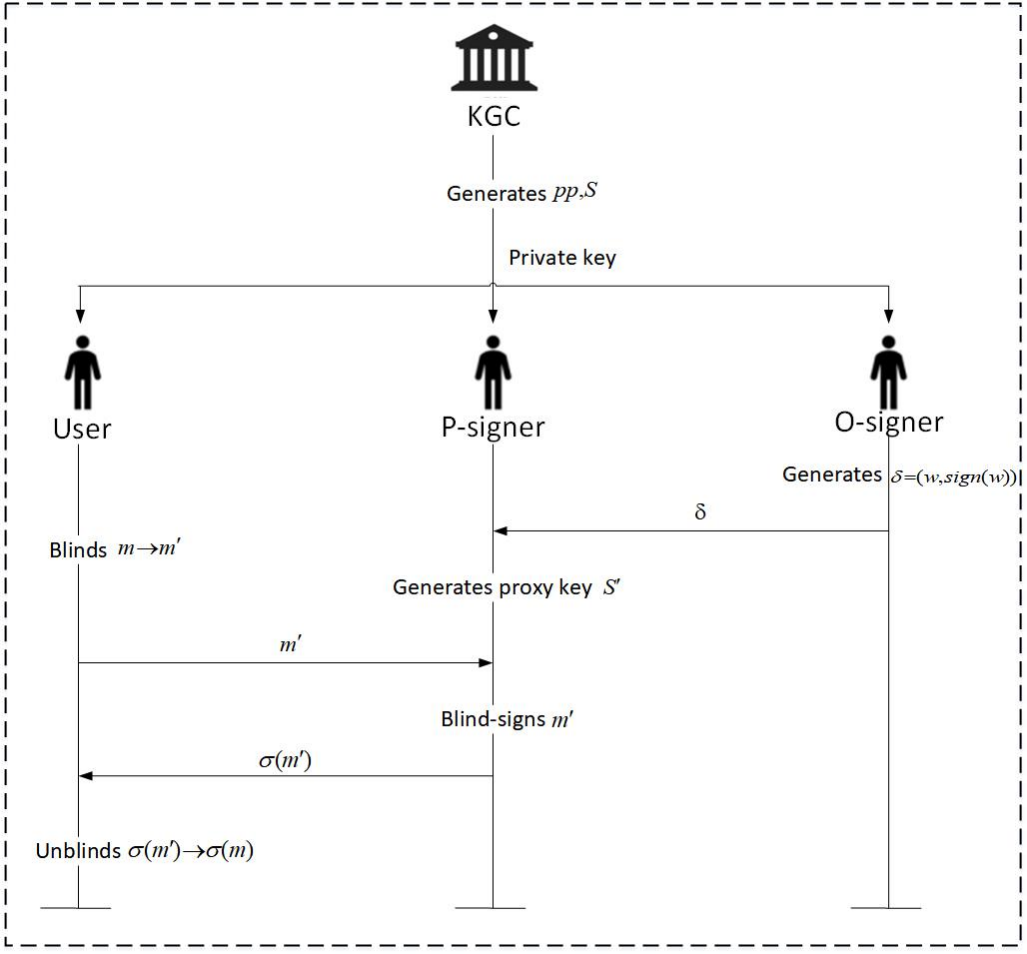
Identity-based proxy blind signature scheme on a lattice.

The ID-Proxy-BS scheme on a lattice proposed in this section is shown in Figure 1. There are six entities in the proposed scheme; they are key generation center KGC, user U, original signer O-signer, proxy signer P-signer, and verifier Verifier. This scheme contains five algorithms; namely, system initialization (Setup), key generation (KeyGen), proxy delegation (ProxyDelegation), proxy key generation (ProxyKeyGen), proxy blind signature (Proxy-BS), and signature verification (Signature Verification). The specific algorithms are as follows.

### 5.1. Setup

The system initialization generates the system public parameters and hash functions using the parameter setting method of the lattice cryptography, and generates the system master public key and master private key using the trapdoor generation algorithm on a lattice. The specific algorithm is below:

(1) Parameter setting: λ denotes the security parameters, q=ploy(n), m=O(nlgq), $u=qI_{n}$, $\sigma \in Z_{q}^{n}$.
(2) Hash function settings: $H:{\left\{0,1\right\}}^{*}\to Z_{2q}^{n\times m}$, $H_{1}:{\left\{0,1\right\}}^{*}\to Z_{2q}^{n\times 3m}$.
(3) KGC runs $TrapGen(1^{\lambda })$ to generate $A\in Z_{2q}^{n\times m}$ and a basis $S\in Z_{2q}^{m\times n}$ of lattice $\Lambda_{2q}^{⊥}\left(A\right)$, where $\left\|S\|\leq O(\sqrt{n\log q}\right)$.
(4) The public parameter is set to $pp=\{A,H,H_{1}\}$; the master private key is msk=S.

### 5.2. KeyGen

In this section, the master public key and the user identity are cascaded to construct the user public key, and the user’s private key is generated through the preimage sampling algorithm on the lattice. The identities of the original signer O-signer and the proxy signer P-signer are $ID_{p}$ and $ID_{o}$, respectively. The specific algorithm is below:

KGC selects the identity $ID_{o}$ and $ID_{p}$, KGC uses the system’s master key to run $S_{o}\in Z_{2q}^{2m\times n}\leftarrow \mathrm{SamplePre}\left(A\|H\left(ID_{o}\right),S,u,\sigma \right)$ such that $\left[A\|H\left(ID_{o}\right)\right]S_{o}=qI_{n}\left(\mathrm{mod}\;2q\right)$ where $\|S_{o}\|\leq \sigma \sqrt{2m}$. Similarly, KGC runs $S_{p}\leftarrow \mathrm{SamplePre}\left(A\|H\left(ID_{p}\right),S,u,\sigma \right)$. The private keys of O-signer and P-signer are $S_{o}$ and $S_{p}$, respectively.

### 5.3. ProxyDelegation

The proxy delegation algorithm completes the authorization of O-Signer’s signature to P-Signer by generating authorization information through the preimage sampling algorithm on the lattice to sign the authorization certificate. Without loss of generality, this section assumes an authorization certificate, which includes the identity of O-signer, the ID of P-signer, and the proxy authorization period. The specific process is as follows:

(1) After O-signer determines the object for P-signer to authorize, O-signer generates an authorization certificate ω and publishes it.
(2) O-signer runs the algorithm $\delta_{1}\leftarrow \mathrm{SamplePre}(A\left|\right|H\left(ID_{o}\right),S_{o},u,H(\omega ))$, where $\delta_{2}\;=\;\omega$. O-signer will send authorization information $\delta \;=\;(\delta_{1},\delta_{2})$ to P-signer.

### 5.4. ProxyKeyGen

In this section, P-signer generates a proxy key based on the authorization information sent by O-signer through the preimage sampling algorithm on the lattice. The specific algorithm is below:

(1) After P-signer receives δ, it verifies that the equation $[A||H\left(ID_{o}\right)]\delta_{1}=qI_{n}\left(\mathrm{mod}\;2q\right)$ holds. If the equality holds, P-signer accepts the authorization; otherwise, O-signer re-authorizes.
(2) If Equation (1) holds, P-signer runs $\mathrm{SamplePre}(A||H\left(ID_{o}\right)\|H\left(ID_{p}\right),S_{p},u,\delta_{2})$ to generate a proxy key $S^{\prime }\in Z_{2q}^{3m\times n}$ such that $[A∥H\left(ID_{q}\right)∥H\left(ID_{p}\right)]S^{\prime }=qI_{n}\left(\mathrm{mod}2q\right)$ and $∥S^{\prime }∥\leq \sigma \sqrt{3m}$.

### 5.5. Proxy-BS

The Proxy-BS algorithm first generates random blinding factors to hide the original message through a bimodal Gaussian distribution, then signs the blinded message through P-signer ’s private key and the proxy key, and finally obtains the signature of the original message by removing the blinding factor. This section includes three stages; namely, blinding, proxy blind signature, and unblinding. The specific algorithm is below:

Before the blinding phase, P-signer randomly selects two vectors $r_{1}\leftarrow D_{\sigma_{2}}^{2m}$, $r_{2}\leftarrow D_{\sigma_{2}}^{3m}$ and computes commitment $x_{1}\leftarrow \left[A\|H\left(ID_{p}\right)\right]r_{1}$, $x_{2}\leftarrow [A\|H\left(ID_{o}\right)\left\|H\left(ID_{p}\right)\right]r_{2}$ to *U*.

#### 5.5.1. Blinding

If a signature is required, user U uses P-signer ’s commitment $x_{1}$, $x_{2}$, blinding factor $y_{1}$, $y_{2}$, and message *m* to hash to complete the blinding process. Then, *U* sends a blind message to P-signer. It is known that *m* is the message to be blinded. The specific algorithm is as follows:

(1) U randomly selects two blinding factors $y_{1}\leftarrow D_{\sigma_{3}}^{2m}$, $y_{2}\leftarrow D_{\sigma_{3}}^{3m}$.
(2) U calculates $c_{1}\leftarrow H_{1}(x_{1}+[A||H\left(ID_{p}\right)]y_{1}\;\mathrm{mod}\;2q,m)$, $c_{2}\leftarrow H_{1}(x_{2}+[A\left|\right|H\left(ID_{o}\right)\left|\right|$ $H\left(ID_{p}\right)]y_{2}\;\mathrm{mod}\;2q,m)$.
(3) U selects a bit b∈{0,1}.
(4) U computes the blinded message $\mu_{1}\leftarrow {\left(-1\right)}^{b}c_{1}$, $\mu_{2}\leftarrow {\left(-1\right)}^{b}c_{2}$.
(5) U sends blind message $\left(\mu_{1},\mu_{2}\right)$ to P-signer.

#### 5.5.2. Proxy Blind Signature

P-signer signs the received blind message $\left(\mu_{1},\mu_{2}\right)$ according to the parameters generated by the preimage sampling algorithm on the lattice. P-signer uses random vector $r_{1}$, $r_{2}$, own private key, and proxy key to perform a proxy blind signature and sends the signature $\left(z_{1},z_{2}\right)$ to *U*. The specific algorithm is as follows:

(1) P-signer uses the random vector selected when generating the commitment for the user $r_{1}\leftarrow D_{\sigma_{2}}^{2m}$, $r_{2}\leftarrow D_{\sigma_{2}}^{3m}$.
(2) P-signer calculates the signature $z_{1}\leftarrow r_{1}+\mu_{1}S_{p}$, $z_{2}\leftarrow r_{2}+\mu_{2}S^{\prime }$ of the blind message $\left(\mu_{1},\mu_{2}\right)$.
(3) P-signer returns the blind signature $\left(z_{1},z_{2}\right)$ to *U*.

#### 5.5.3. Unblinding

User U receives the blind signature $\left(z_{1},z_{2}\right)$ from P-signer and U unblinds the signature to recover the signature of the message *m*. The specific steps are as follows:

(1) U uses the blinding factor $y_{1}\leftarrow D_{\sigma_{3}}^{2m}$, $y_{2}\leftarrow D_{\sigma_{3}}^{3m}$ selected in the blinding message phase.
(2) U calculates the signature $e_{1}\leftarrow y_{1}+z_{1}$, $e_{2}\leftarrow y_{2}+z_{2}$ of the original message *m*.

### 5.6. Signature Verification

The signature $\left(e_{1},e_{2}\right)$ is verified based on the public key of P-signer and O-signer, and the hash values $c_{1}$ and $c_{2}$ are generated by the user during the blinding. If the signature matches the conditions, it is accepted; otherwise, it is rejected. The signature verification algorithm is shown below:

(1) $\|e_{1}\|\leq B_{1}$, $\|e_{2}\|\leq B_{2}$ (where $B_{1}=\eta \sqrt{2m\sigma }$, $B_{2}=\eta \sqrt{3m\sigma }$, η∈[1.1,1.4]).
(2) ${∥e_{1}∥}_{\infty }\leq q/4$, ${∥e_{2}∥}_{\infty }\leq q/4$.
(3) $c_{1}=H_{1}\left(\left[A\left|\right|H\left(ID_{p}\right)\right]e_{1}+qc_{1}\;\mathrm{mod}\;2q,m\right)$.
(4) $c_{2}=H_{1}([A\left|\right|H\left(ID_{o}\right)||H\left(ID_{p}\right)]e_{2}+qc_{2}\;\mathrm{mod}\;2q,m)$.

If conditions (1), (2), (3), and (4) are met, the signature is valid; otherwise, the signature is invalid.

## 6. Performance Analysis

### 6.1. Correctness

In this section, we give proof of correctness for the ID-Proxy-BS scheme on a lattice. When receiving the signature $\left(e_{1},e_{2}\right)$, $\left(c_{1},c_{2}\right)$, the Verifier first runs the signature verification algorithm to verify that the signature is valid. It judges the four conditions $\|e_{1}\|\leq B_{1}$, $\|e_{2}\|\leq B_{2}$, ‖e1‖∞≤qq44, ‖e2‖∞≤qq44; if any one of them is not met, the signature is invalid. Otherwise, according to the public key of P-signer and O-signer and the hash value $\left(c_{1},c_{2}\right)$ generated by the user during the blinding, the Verifier verifies whether the following two equations are true. The details are as follows:

(1) The Verifier verifies that equation $c_{1}=H_{1}\left(\left[A\left|\right|H\left(ID_{p}\right)\right]e_{1}+qc_{1}\;\mathrm{mod}\;2q,m\right)$ holds:
  (5) $$\begin{array}{c} {}[A||H\left(ID_{p}\right)]e_{1}+qc_{1}=[A||H\left(ID_{p}\right)]\left(y_{1}+z_{1}\right)+qc_{1}\\ =[A||H\left(ID_{p}\right)]y_{1}+[A||H\left(ID_{p}\right)]z_{1}+qc_{1}\\ =[A||H\left(ID_{p}\right)]\left(r_{1}+\mu_{1}S_{p}\right)+[A||H\left(ID_{p}\right)]y_{1}+qc_{1}\\ =[A||H\left(ID_{p}\right)]r_{1}+{\left(-1\right)}^{b}[A||H\left(ID_{p}\right)]S_{p}c_{1}+[A||H\left(ID_{p}\right)]y_{1}+qc_{1}\\ =x_{1}+{\left(-1\right)}^{b}qc_{1}+qc_{1}+[A||H\left(ID_{p}\right)]y_{1}\\ =x_{1}+[A||H\left(ID_{p}\right)]y_{1}\left(\mathrm{mod}\;2q\right) \end{array}$$
(2) The Verifier verifies that equation $c_{2}=H_{1}([A\left|\right|H\left(ID_{o}\right)||H\left(ID_{p}\right)]e_{2}+qc_{2}\;\mathrm{mod}\;2q,m)$ holds:
  (6) $$\begin{array}{c} {}[A\left|\right|H\left(ID_{o}\right)||H\left(ID_{p}\right)]e_{2}+qc_{2}=[A\left|\right|H\left(ID_{o}\right)||H\left(ID_{p}\right)]\left(y_{2}+z_{2}\right)+qc_{2}\\ =[A\left|\right|H\left(ID_{o}\right)||H\left(ID_{p}\right)]y_{2}+[A\left|\right|H\left(ID_{o}\right)||H\left(ID_{p}\right)]z_{2}+qc_{2}\\ =[A\left|\right|H\left(ID_{o}\right)||H\left(ID_{p}\right)]\left(r_{2}+\mu_{2}S^{\prime }\right)+[A\left|\right|H\left(ID_{o}\right)||H\left(ID_{p}\right)]y_{2}+qc_{2}\\ =[A\left|\right|H\left(ID_{o}\right)||H\left(ID_{p}\right)]r_{2}+{\left(-1\right)}^{b}[A\left|\right|H\left(ID_{o}\right)||H\left(ID_{p}\right)]S^{\prime }c_{2}+[A\left|\right|H\left(ID_{o}\right)||H\left(ID_{p}\right)]y_{2}+qc_{2}\\ =x_{2}+{\left(-1\right)}^{b}qc_{2}+qc_{2}+[A\left|\right|H\left(ID_{o}\right)||H\left(ID_{p}\right)]y_{2}\\ =x_{2}+[A\left|\right|H\left(ID_{o}\right)||H\left(ID_{p}\right)]y_{2}\left(\mathrm{mod}\;2q\right) \end{array}$$

If (1) and (2) above are valid, the ID-Proxy-BS scheme on a lattice satisfies correctness.

### 6.2. Blindness

**Theorem** **1.** *The ID-Proxy-BS on-lattice scheme proposed in this paper satisfies blindness.*

**Proof.**  An adversary signer *S* cannot obtain useful information from signed messages. Suppose the adversary *S*, having the advantage $A\mathrm{dv}(S^{*})$, interacts with two different users $U_{0}$, $U_{1}$ to attack our scheme.

Setup: We are given a random coin b∈{0,1}, which cannot be known by *S*. $U_{1}$ and $U_{2}$ randomly select two messages $m_{b}$ and $m_{1-b}$, respectively, and send them to *S*.

Signature: After *S* has received the message from $U_{1}$ and $U_{2}$, *S* executes the blind signature algorithm with two users $U_{1}\left(m_{b}\right)$ and $U_{2}\left(m_{1-b}\right)$ simultaneously, and finally $U_{1}$ and $U_{2}$ generate signatures $\sigma (m_{b})$ and $\sigma (m_{1-b})$, respectively, and send them to *S*.

Guess: After *S* has received the signature from $U_{1}$ and $U_{2}$, *S* guesses *b*.

When performing the proxy blind signature algorithm, due to the random variables, we only need to prove the blinded messages μ and (c,e) and note that since *c* is the result of a hash function and is randomly generated, we do not have to account for it. The specific analysis process is as follows:

- The distribution of μ. The interaction of adversary *S* with $\sigma (m_{b})$ and $\sigma (m_{1-b})$, respectively, generates $\mu_{b}$ and $\mu_{1-b}$. The statistical distance of $\mu_{b}$ and $\mu_{1-b}$ is $\Delta =\left(\mu_{s},\mu_{1-b}\right)=\frac{1}{2}\sum_{\tilde{\mu }\in Z^{n}}\left|\mathrm{Pr}\left(\mu_{b}=\tilde{\mu }\right)-\mathrm{Pr}\left(\mu_{1-b}=\tilde{\mu }\right)\right|$. Since $\mu \leftarrow {\left(-1\right)}^{b}c$ and it is output with probability $\min(\frac{D_{\sigma_{1}}^{\mu }\left(\mu \right)}{M_{1},D_{c,\sigma_{1}\left(\mu \right)}^{m}},1)$, $\mu_{b}$ and $\mu_{1-b}$ have the same distribution $D_{\sigma_{1}}^{m}$ through the rejection sampling algorithm. The statistical distance satisfies $\Delta (\mu_{b},\mu_{1-b})=0$, and they are independent of the signed messages, so the adversary S cannot distinguish them.
- The distribution of *e*. Similar to μ because $e_{b}$ and $e_{1-b}$ have the same distribution $D_{\sigma_{2}}^{m}$ through the rejection sampling algorithm. Their statistical distance satisfies $\Delta (e_{b},e_{1-b})=0$ and they are independent of signed messages, so the adversary *S* cannot distinguish them. The final P-signer cannot associate the message with the signatures μ and (c,e).

□

### 6.3. Unforgeability

**Theorem** **2.** *In the random oracle model, the ID-Proxy-BS on-lattice scheme satisfies EUF-CMA security if no adversary $F_{1}$ forges a valid proxy blind signature with a non-negligible advantage ε assuming that the SIS problem is hard.*

**Proof.**  Suppose there is a probabilistic polynomial adversary $F_{1}$ who performs $q_{H}$ hash queries and $q_{s}$ signature queries, and forges a valid proxy blind signature with non-negligible advantage ε. $F_{1}$ outputs the challenge identity ID. The following simulates the interaction between the challenger T and the adversary $F_{1}$.

Hash queries: T maintains an initialized empty list $L_{1}$ to store the hash value of the message *m*. $F_{1}$ inputs *m*. T first checks the corresponding tuple in $L_{1}$. If it exists, T returns (m,H(m)) to $F_{1}$; if not, T chooses $c\leftarrow \{v\in {\left\{-1,0,1\right\}}^{k}:||v|{|}_{1}\leq k\}$ and selects $e\leftarrow D_{\sigma_{3}}^{m}$, with c=H([A||H(ID)]e+qcmod2q,m). T stores (e,c) and returns *c* to $F_{1}$.

Signature queries: T maintains an initialized empty list $L_{2}$ to store the signature of the message *m*. When $F_{1}$ sends a query for the signature of the message *m*, T first checks the corresponding tuple in $L_{2}$. If it exists, T returns (m,c,e) to $F_{1}$; otherwise, T will run the proxy blind signature algorithm to generate the signature pair (c,e) to $F_{1}$.

Forgery: After $F_{1}$ decides to end these queries, $F_{1}$ outputs a forged signature. T will use this forged signature to solve the SIS problem. Suppose $c=c_{j}$. There are two possibilities for $c_{j}$: one is $c_{j}$ generated in the signature queries and the other is generated in the hash queries.

When $c_{j}$ is generated in signature queries, due to the fact that $c=c_{j}$, then $H\left(\left[A\left|\right|H\left(ID\right)\right]e+qc_{j},m\right)=H\left(\left[A\left|\right|H\left(ID\right)\right]e^{\prime }+qc_{j},m^{\prime }\right)$. If $m\neq m^{\prime }$ or $\left[A∥H\left(ID\right)\right]e+qc_{j}\neq \left[A∥H\left(ID\right)\right]e^{\prime }+qc_{j}$, this means that $F_{1}$ has found a preimage of $c_{j}$. Therefore, $m=m^{\prime }$, $\left[A\left|\right|H\left(ID\right)\right]e+qc_{j}=\left[A\left|\right|H\left(ID\right)\right]e^{\prime }+qc_{j}$, and $A(e-e^{\prime })=0\;\mathrm{mod}\;2q\;$. Since $e-e^{\prime }\neq 0$, the SIS problem is solved.

When $c_{j}$ is generated in hash queries, T records the adversary’s forged signatures $\left(e,c_{j}\right)$ on messages *m*, and selects randomly $c_{t}^{\prime },...,c_{t}^{\prime }\leftarrow B^{k}$. According to Lemma [18], the probability that $F_{1}$ generates a new forged signature $\left(e^{\prime },c_{j}^{\prime }\right)\left(c_{j}\neq c_{j}^{\prime }\right)$ is $\left(\varepsilon -\frac{1}{B_{k}^{n}}\right)\left(\frac{q-1/B_{k}^{n}}{q_{s}+q_{H}}-\frac{1}{B_{k}^{n}}\right)$. Since $\left[A\left|\right|H\left(ID\right)\right]e-qc_{j}=\left[A\left|\right|H\left(ID\right)\right]e^{\prime }-qc_{j}^{\prime }$, the public key and the private key satisfy $\left[A\left|\right|H\left(ID\right)\right]S=qI_{n}\;\mathrm{mod}\;2q$; therefore, we can obtain the equation $\left[A\left|\right|H\left(ID\right)\right]\left(e-e^{\prime }\right)=q\left(c_{j}-c_{j}^{\prime }\right)I_{n}\;\mathrm{mod}\;2q$. Since $c_{j}\neq c_{j}^{\prime }$, we can deduce that $e-e^{\prime }\neq 0\;\mathrm{mod}\;2q$. We know $q(c_{j}-c_{j}^{\prime })\;\mathrm{mod}\;q=0$, so $\left[A\left|\right|H\left(ID\right)\right]\left(e-e^{\prime }\right)=0\;\mathrm{mod}\;2q$. It can be seen that we find a non-zero vector *v* with a probability of at least $\beta =(\frac{1}{2}-2^{-100})\left(\varepsilon -\frac{1}{B_{k}^{n}}\right)\left(\frac{q-1/B_{k}^{n}}{q_{s}+q_{H}}-\frac{1}{B_{k}^{n}}\right)$ such that [A||H(ID)]v=0. □

### 6.4. Efficiency Analysis

In this subsection, we present a comparison with the current literature Refs. [13,18,28]. Assuming that the parameters (n,m,d,k,q,σ) in this paper are the same as those in the existing literature, the specific comparison result will show in Table 1. The parameters of the proposed scheme are set as shown in Table 2.

According to Table 1, compared with [18] and [13], the key length and signature length of this scheme are relatively large. The public key length, private key length, and signature length of the ID-Proxy-BS on-lattice scheme are smaller than those in [28].

**Figure 2:**
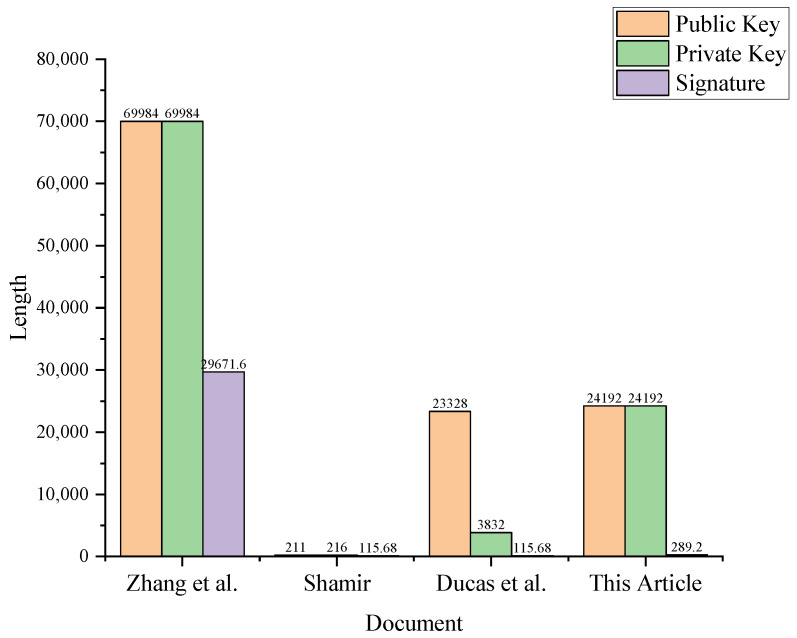
The specific results of the proposed solution compared to the literature Refs. [13,18,28].

In this study, we set the security parameter λ to 128 bits. At the same time, we chose appropriate parameters *n*, *q*, *m* to ensure the security of public and private keys. Since the signature obeys the distribution $D_{\sigma_{3}}^{m}$, the signature of the proposed scheme in this paper is $(5m)\log\left(12\sigma_{3}\right)$ bits. Based on the specific values of these parameters, we provide the comparison results of our scheme with the current schemes, as shown in Figure 2.

## 7. A Quantum-Resistant Proxy E-Voting System

In this section, first, we give the conditions that a secure e-voting system needs to satisfy. Then, we apply the identity-based proxy blind signature (ID-Proxy-BS) on-lattice scheme to e-voting, and design a quantum-resistant proxy e-voting system. Finally, we perform a performance analysis of the proposed e-voting system.

### 7.1. Basic Requirements for E-Voting

E-voting has stimulated people’s research interest due to its advantages of saving time and effort [29]. When building an e-voting system, it is necessary to ensure the privacy of voters and the accuracy of voting. Therefore, an e-voting system should meet the following basic requirements:

(1) Legitimacy: Only legitimate voters who have passed identity verification can vote.
(2) Anonymity: Except for the voter themselves, no one else knows what the voter voted for.
(3) Verifiability: Every voter can verify whether their votes have been counted correctly.

### 7.2. A Quantum-Resistant Proxy E-Voting System

**Figure 3:**
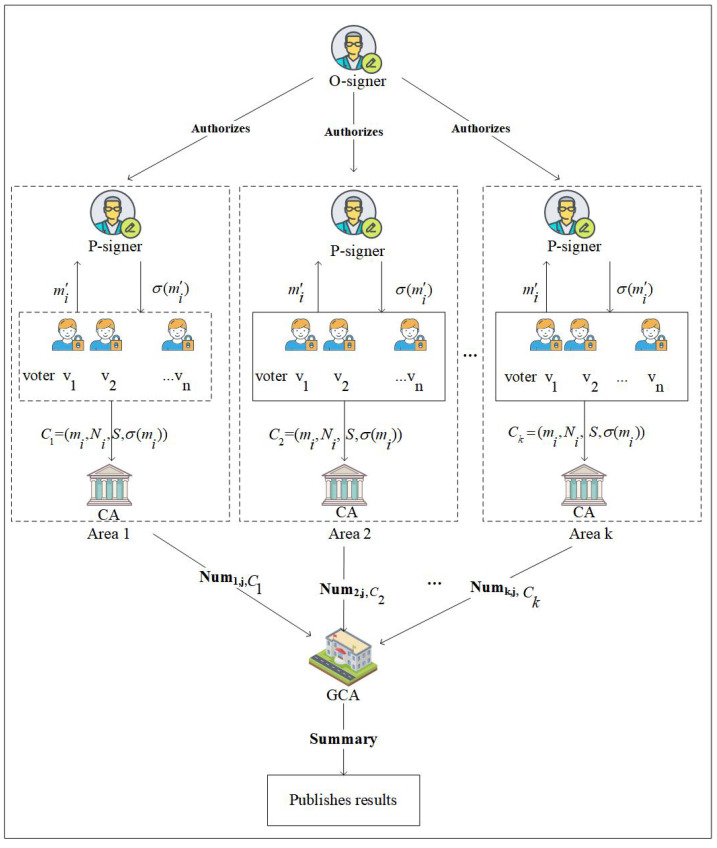
E-voting system based on ID-Proxy-BS on-lattice scheme.

The above-mentioned e-voting does not take into account the quantum security and transmission efficiency of ballots during transmission. Therefore, in this section, we apply the identity-based proxy blind signature (ID-Proxy-BS) on-lattice scheme to e-voting, and propose a multi-region proxy e-voting system that is resistant to quantum attacks. The architecture of the e-voting system is shown in Figure 3. There are *k* constituencies in this system, and each constituency sets up a proxy signature agency and counts votes separately, thereby improving voting efficiency. Second, the voter hides the content of the ballot in the signature, so that the privacy of the voter is protected. Finally, based on the characteristics of the lattice, the proposed e-voting system can resist quantum attacks.

The quantum-resistant proxy e-voting system consists of five entities, which are voters, registration agency, voting agency, counter agency, and general counter agency.

- Voter: A voter; that is, the owner of the content of the ballot.
- Registration agency (RA): The registration agency checks the identity of voters.
- Voting agency: The voting agency signs the voter’s ballot to validate that ballot.
- Counter agency (CA): The counter agency is responsible for counting the number of votes in the constituency.
- General counter agency (GCA): The General counter agency is responsible for counting the total votes and publishing the results.

Specifically, the proposed e-voting system in this paper mainly includes four stages: setup, vote writing stage, voting stage, and vote counting stage. Table 3 shows the symbols and definitions used in this system.

#### 7.2.1. Setup

Let λ be the security parameters, q=ploy(n), m≥2nlgq. Hash function $H:{\left\{0,1\right\}}^{*}\to Z_{2q}^{n\times m}$. First, the registration agency RA runs $\left(A,S\right)\leftarrow TrapGen\left(1^{n}\right)$ to generate the system’s master public key *A* and master private key *S*. Then, the RA runs $\mathrm{SamplePre}(A\|H\left(ID_{i}\right),S,u,\sigma )$ to generate the user’s private key. It is known that the public and private key pairs of O-signer and P-signer are $\left(ID_{o},S_{o}\right)$ and $\left(ID_{P},S_{P}\right)$, respectively. Finally, the RA is responsible for registering every legal voter. The specific process is as follows:

(1) The RA publishes a list of voters and sends the registration form RF to voter $V_{i}$ who is on the list.
(2) $V_{i}$ runs $x_{i}\leftarrow \mathrm{SamplePre}\left(A\|H\left(ID_{i}\right),S_{i},\sigma \right)$, then $V_{i}$ fills in $\left(ID_{i},x_{i}\right)$ on RF, and sends RF to the RA.
(3) The RA receives the RE completed by $V_{i}$; the RA uses $V_{i}$’s public key to verify the legitimacy of $V_{i}$’s identity. If $\left[A\|H\left(ID_{i}\right)\right]x_{i}=qI_{n}\;\mathrm{mod}\;2q$ and $\|x_{i}\|\leq \sigma \sqrt{2m}$, the RA randomly selects a ballot number $N_{i}\in {\left\{0,1\right\}}^{*}$ for $V_{i}$, and runs $X_{i}\leftarrow \mathrm{SamplePre}(A\|H\left(ID_{i}\right)\left|\right|N_{i},S,\sigma )$. The RA sends $\left(ID_{i},N_{i},X_{i}\right)$ to $V_{i}$.
(4) After $V_{i}$ receives $\left(ID_{i},N_{i},X_{i}\right)$, $V_{i}$ uses the RA’s public key to verify the legitimacy of the ballot. If $AX_{i}=qI_{n}\;\mathrm{mod}\;2q$ and $\|X_{i}\|\leq \sigma \sqrt{3m}$, $V_{i}$ accepts the ballot number; otherwise, $V_{i}$ re-applies to the RA for the ballot number.

#### 7.2.2. Vote Writing Stage

Suppose there are *n* voters $V_{1},V_{2},\cdots V_{n}$ and *m* candidates $C_{1},C_{2},\cdots C_{m}$. If $V_{i}$ wants to vote for candidate $C_{j}$, it is recorded as $m_{i}\left[j\right]=1;$ otherwise, $m_{i}\left[j\right]=0$. $V_{i}$ fills in the ballot as $m_{i}=m_{i}\left[1\right]m_{i}\left[2\right]\cdots m_{i}\left[m\right]$.

#### 7.2.3. Voting Stage

In the voting stage, O-signer grants their signing rights to the P-signers of each constituency, and the P-signers of each constituency sign the blinded ballots in the areas under their jurisdiction.

(1) Proxy delegation
  After O-signer determines the object P-signer to authorize, it runs ProxyDelegation $\left(A,H\left(ID_{o}\right),S_{o},\omega \right)$ to generate authorization information $\delta \;=\;(\delta_{1},\delta_{2})$ and sends it to P-signer. After P-signer receives δ, it verifies $[A||H\left(ID_{o}\right)]\delta_{1}=qI_{n}\left(\mathrm{mod}\;2q\right)$ whether it is established. If the equality is established, P-signer accepts the authorization, otherwise, O-signer re-authorizes.
(2) Proxy key generation
  If the authorization is successful, P-signer runs $\mathrm{SamplePre}(A||H\left(ID_{o}\right)\|H\left(ID_{p}\right),$ $S_{p},u,\delta_{2})$ to generate a proxy key $S^{\prime }\in Z_{2q}^{3m\times n}$.
(3) Blind signature generation
  ① $V_{i}$ runs the blinding algorithm to obtain blinded ballots $\left(\mu_{1},\mu_{2}\right)$ of $m_{i}$ and send $\left(\mu_{1},\mu_{2}\right)$ to P-signer.
  ② P-signer signs the blinded ballot $\left(\mu_{1},\mu_{2}\right)$ to obtain blinded signature $\left(z_{1},z_{2}\right)$ and sends $\left(z_{1},z_{2}\right)$ to $V_{i}$.
  ③ $V_{i}$ unblinds the signature $\left(z_{1},z_{2}\right)$ to obtain $\left(e_{1},e_{2}\right)$. $\left(m_{i},N_{i},S,e_{1},e_{2}\right)$ is the proxy blind signature of the ballot.

#### 7.2.4. Counting Stage

The voter $V_{i}$ sends signed ballots $\left(m_{i},N_{i},S,e_{1},e_{2}\right)$ to the counting agency CA. The CA verifies the legitimacy and uniqueness of the ballot; that is, the CA verifies whether ①–④ are established at the same time:

① $\|e_{1}\|\leq B_{1}$, $\|e_{2}\|\leq B_{2}$ (where $B_{1}=\eta \sqrt{2m\sigma }$, $B_{2}=\eta \sqrt{3m\sigma }$, η∈[1.1,1.4]).

② ${∥e_{1}∥}_{\infty }\leq q/4$, ${∥e_{2}∥}_{\infty }\leq q/4$.

③ $c_{1}=H_{1}\left(\left[A\left|\right|H\left(ID_{p}\right)\right]e_{1}+qc_{1}\;\mathrm{mod}\;2q,m\right)$.

④ $c_{2}=H_{1}([A\left|\right|H\left(ID_{o}\right)||H\left(ID_{p}\right)]e_{2}+qc_{2}\;\mathrm{mod}\;2q,m)$.

If the verification passes and the ballot number $N_{i}$ is unique, the CA accepts the ballot; otherwise, the CA discards it. After the voting is completed, the CA first calculates the voting results of all voters $V_{i}$ for each $C_{j}$; then, the CA calculates the number of votes $m_{1}\left[j\right]+m_{2}\left[j\right]+\cdots +m_{n}\left[j\right]$ for each $C_{j}$. Finally, the CA of each constituency sends the number of votes $Num_{k,j}$ of $C_{j}$ and signed ballots $C_{k}=\left(m_{i},N_{i},S,e_{1},e_{2}\right)$ to GCA to summarize and publish the voting results.

### 7.3. Performance Analysis

The e-voting system proposed in this paper has the following characteristics.

(1) Legality. Before voting, every voter must be registered and verified by the RA before becoming a legal voter. In the registration phase, the voter $V_{i}$ registers using their own identity $ID_{i}$ and signs with their own private key, i.e., $\left(ID_{i},x_{i}\right)$. Even if an adversary fills in the registration information to pretend to be a voter, they cannot know the private key $S_{i}$ of the voter. Since the SIS problem is a hard problem, the adversary cannot forge $x_{i}$ to be a legitimate voter.
(2) Anonymity. In the voting stage, $V_{i}$ can obtain P-signer’s blind signature through the ID-Proxy-BS scheme. Therefore, the e-voting system proposed in this paper enables anonymous voting by voters, and no one can associate the vote with the voter except the voter themselves.
(3) Efficiency: In the e-voting system proposed in this paper, O-signer grants signature rights to the P-signer for each constituency by region, and the P-signers for each constituency sign the blinded ballots for the region under their jurisdiction at the same time, thus increasing the efficiency of voting.
(4) Verifiability. ① In the registration stage, $V_{i}$ obtains the unique ballot number $N_{i}$. ② The total number of signed ballots $\left(m_{i},N_{i},S,e_{1},e_{2}\right)$ and the total number of ballots $m_{1}\left[j\right]+m_{2}\left[j\right]+\cdots m_{n}\left[j\right]$ of $C_{j}$ published on the electronic bulletin board by the CA can be used by voters to verify that the ballot papers have been counted.

In the e-voting system proposed in this section, voters hide the content of the ballot in their signatures and realize anonymous voting. In large-scale elections, setting up agencies for each district improves the efficiency of e-voting. Based on the characteristics of a lattice, the proposed e-voting system can resist quantum attacks. Therefore, the e-voting system proposed in this paper is anonymous, efficient, and resistant to quantum attacks.

## 8. Conclusions

In this paper, to simplify key management and resist quantum attacks, we have proposed a post-quantum secure identity-based proxy blind signature (ID-Proxy-BS) scheme on a lattice using a matrix cascade technique and lattice cryptosystem. In the proposed scheme, firstly, we cascaded the user identity and the master public key to construct the public key of the lattice signature, and generated random parameters through a bimodal Gaussian distribution and rejection sampling algorithm. Then, the security of the ID-Proxy-BS scheme was proved based on the SIS problem under the ROM. Finally, we applied the scheme to e-voting, and designed a quantum-resistant proxy e-voting system. The system enables multi-regional electronic voting and satisfies anonymity, high efficiency, and anti-quantum attack.

## Figures and Tables

**Table 1:** Comparison of the proposed solution w.r.t. to the state-of-the-art.

Document	Public Key Length	Private Key Length	Signature Length
[18]	3mnlogq	3mnlogq	(mn+dm)log(12σ)
[28]	mnlog(2d+1)	nklogq	2mlog(12σ)
[13]	mnlogq	mklogq	2mlog(12σ)
This article	mnlog(2q)	mnlog(2q)	(5m)log(12σ)

**Table 2:** Parameter settings.

Parameter	Value
*n*	512
*q*	$2^{27}$
*m*	13,824
*d*	1
λ	128
$\sigma_{1}$	64
$\sigma_{2}$	$2^{20}$
$\sigma_{3}$	$2^{30}$
Signature length	289.2 KB
Secret key length	24,192 KB
Public key length	24,192 KB

**Table 3:** Symbol definition.

Symbol	Definition
$V_{i}$	Voters
$ID_{i}$	Identity
RF	Registration form
$m_{i}$	Content of the ballot
RA	Registration agency
O-signer	Original signer
P-signer	Proxy signer
CA	Counting agency
GCA	Tallying agency

## Data Availability

Not applicable.
